# Perceptual salience affects the contents of working memory during free-recollection of objects from natural scenes

**DOI:** 10.3389/fnhum.2015.00060

**Published:** 2015-02-17

**Authors:** Tiziana Pedale, Valerio Santangelo

**Affiliations:** ^1^Department of Psychology, Sapienza University of RomeRome, Italy; ^2^Cognitive Neuroscience Group, Neuroimaging Laboratory, Santa Lucia FoundationRome, Italy; ^3^Department of Philosophy, Social, Human and Educational Sciences, University of PerugiaPerugia, Italy

**Keywords:** visual, salience, working memory, capacity, free recollection, objects, natural scenes

## Abstract

One of the most important issues in the study of cognition is to understand which are the factors determining internal representation of the external world. Previous literature has started to highlight the impact of low-level sensory features (indexed by saliency-maps) in driving attention selection, hence increasing the probability for objects presented in complex and natural scenes to be successfully encoded into working memory (WM) and then correctly remembered. Here we asked whether the probability of retrieving high-saliency objects modulates the overall contents of WM, by decreasing the probability of retrieving other, lower-saliency objects. We presented pictures of natural scenes for 4 s. After a retention period of 8 s, we asked participants to verbally report as many objects/details as possible of the previous scenes. We then computed how many times the objects located at either the peak of maximal or minimal saliency in the scene (as indexed by a saliency-map; Itti et al., 1998) were recollected by participants. Results showed that maximal-saliency objects were recollected more often and earlier in the stream of successfully reported items than minimal-saliency objects. This indicates that bottom-up sensory salience increases the recollection probability and facilitates the access to memory representation at retrieval, respectively. Moreover, recollection of the maximal- (but not the minimal-) saliency objects predicted the overall amount of successfully recollected objects: The higher the probability of having successfully reported the most-salient object in the scene, the lower the amount of recollected objects. These findings highlight that bottom-up sensory saliency modulates the current contents of WM during recollection of objects from natural scenes, most likely by reducing available resources to encode and then retrieve other (lower saliency) objects.

## Introduction

When we look at a complex scene for a small amount of time we will probably remember only some of the information that was included in the original scene. The possibility of remembering this information is strictly related to the chances of building an internal (memory) representation of the scene. Although internal representations are crucial for a number of high-level cognitive processes (e.g., Fuster, 2006), it is still not entirely clear why some objects in a scene have more chance than others to be stored in memory (see, for reviews, Gazzaley and Nobre, 2012; Kiyonaga and Egner, 2013). Previous literature provided evidence that highlight the key role played by low-level sensory features (i.e., line orientation, intensity contrast and color opponency, as indexed by saliency-maps; Itti et al., 1998) in biasing attention selection and working memory (WM) encoding (Stirk and Underwood, 2007; Fine and Minnery, 2009; Melcher and Piazza, 2011; Santangelo and Macaluso, 2013; Spotorno et al., 2013; see, for a recent review, Santangelo, 2015).

For instance, Fine and Minnery (2009) conducted a behavioral study in which they asked participants to remember the position of 3–5 target icons placed on a geographical map (encoding phase). After a retention interval, participants were asked to relocate the icons either on the map (50% of trials) or on a blank screen (50% of trials). Irrespective of the retrieval condition (map-on vs. map-off), Fine and Minnery found that the more salient an icon was (quantified using Itti et al., 1998, model), the more accurate subjects were in repositioning the icons. These findings provided initial evidence about the impact of low-level sensory features on the encoding of objects in WM. Consistent findings were also reported by Santangelo and Macaluso (2013) using a delayed match-to-sample task during viewing of natural scenes. During fMRI scanning, participants were presented with natural scenes for 4 s (encoding phase), which were followed by a retention interval of 8 s. After that, participants judged the location (same/different) of a target-object extracted from the initial scene. Santangelo and Macaluso found that retrieval accuracy increased along with object saliency at encoding, indicating that the probability of WM encoding was a function of sensory salience.

Overall, this literature consistently demonstrated that bottom-up sensory salience increases the probability of an object to be successfully selected, and then stored in memory. Interestingly, recent evidence suggests that the role of perceptual saliency might not only affect the storage of single objects (according to their specific saliency level), but the overall content of the WM representation. Melcher and Piazza (2011) reported a series of experiments in which they manipulated bottom-up sensory salience of simple stimuli. For the memory set, they presented displays including a variable number (i.e., a variable set size) of Gabor patches with different orientations for 200 ms. The saliency of one Gabor was manipulated by increasing its contrast and/or size. After a delay of 1000 ms a test Gabor was presented. Participants were asked to judge whether the orientation of the test Gabor was the same or different compared to the Gabor at the same location in the memory set. Melcher and Piazza found that memory performance for the most salient Gabor remained high, irrespective of increased set size, while memory performance dropped dramatically with set size when a non-salient item was tested. This finding was interpreted by Melcher and Piazza as evidence that the overall WM capacity was influenced by changes in the relative saliency of the items.

A similar conclusion was reached by Pooresmaeili et al. (2014). In each trial, they presented a tilted bar as a memory sample. Participants had to keep in mind the orientation of this bar for a following memory-based choice. In the next display, a bar with the same orientation as the sample bar and a bar with a different orientation were presented on either side of a central fixation point. On some trials, Pooresmaeili et al. manipulated the saliency of either the bar matching or not-matching with the sample bar (changing its color to red), while in the remaining trials all the bars were displayed in white (no salience condition). In Exp. 1, participants were asked to find the test bar that matched the orientation of the sample; while in Exp. 2, they had to find the non-matching bar. Pooresmaeili et al. reported that their participants chose a visually salient item more often when they looked for matching features and less often when they looked for a non-match, indicating that salient items are more likely to be identified as a match. Pooresmaeili et al. interpreted this finding in terms of capacity limitations during the test phase, in which the visually salient item is more likely to consume WM resources, with the effect to be erroneously identified as matching with the memory sample.

These studies provided intriguing evidence linking perceptual saliency to the modulation of available WM capacity. Here we further investigate this issue using more complex stimuli, i.e., pictures representing natural scenes. Natural scenes typically included multiple objects, which entail a high-level of stimulus competition during attention selection and access in memory (see, e.g., Henderson and Hollingworth, 1999; Henderson, 2003; Hollingworth, 2012). We therefore aim to assess whether WM capacity can be modulated by perceptual saliency (cf. Melcher and Piazza, 2011; Pooresmaeili et al., 2014) also when using complex and ecologically-valid material, i.e., complex and natural scenes. For this, we presented pictures of natural scenes for 4 s. After a retention period of 8 s, we asked participants to verbally report as many objects/details as possible of the previous scenes (i.e., a free recollection task; e.g., Standing, 1973). We then computed how many times the objects located at either the peak of maximal- or minimal-saliency in the scene (as indexed by a saliency-map; Itti et al., 1998) were recollected by participants. This procedure allowed us to compute two different indexes related to maximal- and minimal-saliency objects, namely “recollection probability” and “recollection position” (i.e., the probability of recollecting that object and its position in the stream of reported items, respectively). If the selection and storage of maximal-saliency objects is facilitated, we would expect higher recollection probability for maximal- compared to minimal-saliency objects. Similarly, if perceptual salience affects the access to scene representation, we would expect that maximal-saliency objects were recollected earlier than minimal-saliency objects.

These indexes (recollection probability and recollection position) were also used to assess the impact of bottom-up sensory saliency in affecting the contents of WM by means of two regression models, one for each saliency condition (maximal or minimal). The choice to use free recollection was motivated by the possibility of measuring WM capacity in a natural context, in terms of the “amount of recollected objects” within each scene. Accordingly, in the first regression model we assessed whether the probability of recollecting maximal-saliency objects (i.e., the recollection probability index) affected the contents of WM, i.e., the overall amount of information successfully reported by participants for each scene. We would expect that the higher the probability of encoding and then recollecting the maximal-saliency object, the more the decrease in the overall amount of reported information. This would indicate that bottom-up sensory saliency affects WM contents, with the storage of the most-salient object in the scene reducing the available resources to store and then recollect other—lower saliency—objects (cf. Melcher and Piazza, 2011). Within the same regression model we also assessed whether the position in which the maximal-saliency object was recollected (i.e., the recollection position index) affected the amount of successfully reported information. This would suggest that the impact of saliency on WM specifically arises during the access to the scene representation stored in WM: the earlier the maximal-saliency object is reported, the smaller the amount of recollected information, indicating that the access to the memory representation for the most-salient object in the scene decreases resource availability to report other—lower saliency—objects. By contrast, a null effect in this latter analysis (i.e., no impact of the recollection position index on the amount of recollected information) would be consistent with the notion that bottom-up saliency mainly affect the encoding—more than retrieval—of objects from natural scenes, in line with previous findings (Santangelo and Macaluso, 2013). Finally, the second regression model assessed the influence of recollection probability and recollection position on the amount of successfully reported information, but now specifically for minimal-saliency objects. We would expect no significant effects for this analysis, indicating that objects associated with low-levels of bottom-up saliency are not attentional capturing and then ineffective in modulating WM contents.

## Methods and materials

### Participants

Twenty healthy volunteers (9 males; mean age = 24.2 years, ranging from 21 to 34 years), students at the University of Perugia, participated in the study. They all gave written informed consent and were naïve to the main purpose of the study.

### Stimuli and task

The set of stimuli consisted of one hundred pictures depicting scenes of everyday life. These images were collected on the World Wide Web and had already been used by Santangelo and Macaluso (2013). The pictures included internal (e.g., a kitchen, a bathroom, etc.) and external scenes (e.g., a garden, a street, etc.), but no single-object photo or living things such as people or animals.

**Figure 1:**
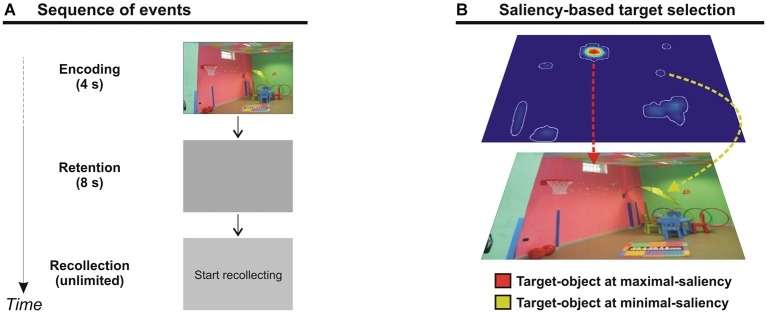
**(A)** Diagram showing the sequence of events during one trial. The trial began with a picture presented for 4 s (encoding phase). A blank display was then shown for 8 s (delay phase), and was followed by a “start recollecting” signal. Participants had no time constraint to recollect as much objects/details as possible from the previous scene. When the recollection was over, participants pressed the space bar for the next trial. **(B)** Selection of target-objects corresponding to the point of maximal- (red line) or minimal-saliency (yellow line) in the scene.

The task consisted in an encoding phase (4 s), a maintenance phase (8 s delay), and a recollection phase (time unlimited) (see Figure 1A). During the encoding phase, participants were presented with a picture, displayed at 18 × 12° of visual angle. Participants were required to memorize as many details as possible for later recollection. In fact, following the 8 s delay (blank screen), a display with the signal “start recollecting” was presented, and participants were asked to report verbally as many objects/details as possible of the previous scene. Participants were instructed to be as accurate as possible, taking all the time they needed (i.e., no time constraint in the recollection phase). When their recollection was over, participants pressed the space bar to move to the next trial. After an inter-trial interval of 1 s a new scene was presented. The order of trials was randomized across participants. Participants’ verbal responses were recorded with an external microphone and digitalized into .wav files.

### Data analysis

Each picture has been analyzed with the Saliency Toolbox 2.2,^1^ which computes saliency maps using local discontinuities in line orientation, intensity contrast, and color opponency (Itti et al., 1998). Using the saliency map, we designated for each picture two “target” objects, corresponding either to the point of maximal-salience of the scene (i.e., the maximal-saliency object) or to the point of minimal-salience of the scene (i.e., the minimal-saliency object; see also Figure 1B). To avoid any ambiguity in selecting the maximal- or minimal-saliency objects within each scene, we excluded those objects (typically, large objects) located over more than one peak of saliency. In fact, it would be unclear in this case which value of saliency should be assigned to that object. This procedure therefore allowed us to be more confident about the contribution of saliency on object memory, computing retrieval performance (see below) associated with clear levels of saliency. Twenty-nine pictures of the initial set were excluded from further analyses, because it was impossible to select within these scenes objects located over one single peak of either maximal or minimal saliency. Importantly, in the final set of pictures there was a significant difference between the average saliency score for maximal- (2.36) and minimal-saliency (0.27) objects (*t*_(70)_, *p* < 0.001). As a final constraint, we made sure that the size of target-objects did not significantly differ between maximal- and minimal-salience conditions (*t*_(70)_; *p* = 0.526).

Participants’ verbal responses were tabulated into a datasheet. Objects in a scene were coded as successfully recollected only when correctly named. When the scene included a number of similar objects (e.g., several “cups” of different colors), an object was assigned as successfully recollected only when it was possible to establish univocally object/name relation (e.g., the recollection of a “green” or a “red” cup). Among the objects recollected by each participant within each scene we searched for the target-object, designated according to maximal- vs. minimal-level of perceptual saliency (see above).

**Figure 2:**
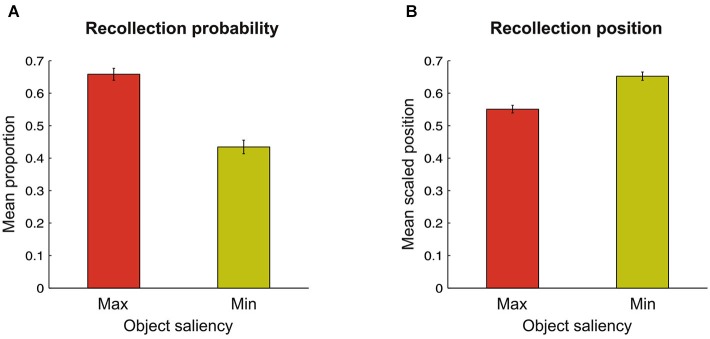
**(A)** Bar graph showing a higher probability to recollect objects corresponding at the location of maximal (red bar) as compared to minimal (yellow bar) saliency in the scene. **(B)** Average position in the streams of recollected objects, indicating that objects corresponding at locations of maximal saliency were reported earlier than objects at minimal saliency. In both graphs, the error bars represents the standard error of the mean.

For each scene and for each participant we computed whether the target-objects (at maximal- or minimal-saliency locations) were successfully recollected, and, if this was the case, what were their positions in the stream of recollected items. This procedure allowed us to compute the mean recollection probability (see Figure 2A) and the mean recollection position (see Figure 2B) of maximal- and minimal-saliency objects. The target-object position in the stream of recollected objects was scaled by the total amount of objects recollected for that scene by that participant (i.e., recollection position index = target-object position / total amount of recollected objects). This weighting procedure allowed us to compare more accurately the meaning of the different target positions among them: for instance, a target-object position of four when twelve objects were recollected has an entirely different meaning compared to when only four objects were recollected, i.e., among the first positions or the last position, respectively. This index varied between 0 and 1: the closer it was to 0, the more the target-object was recollected among the first positions; by contrast, the closer it was to 1, the more the target-object was recollected among the last positions.

Finally, we computed the amount of recollected objects for each scene by each participant when either the maximal- or minimal-saliency object was successfully recollected (i.e., when the target-object was part of the internal memory representation of the scene). This amount was now scaled by the average amount recollected by all participants in that scene (i.e., recollected amount = number of objects recollected by that participant/average amount of objects recollected by all participants). Again, this weighting procedure allowed us to compare more accurately the meaning of each amount among them: for instance, to recollect six objects in a scene in which the whole group recollected an average of twelve objects is a poor performance, but recollecting six objects when the group recollected an average of five is an excellent performance. The closer this index was to 1, the closer the single subject performance was to the group average; the more this index was distant from 1, the more the performance was distant to the group average (i.e., poorer performance <1; better performance >1). Averaging across participants, we obtained the mean scaled amount of recollected objects for each single scene.

To assess the impact of perceptual saliency on WM contents we used two regression analyses, one for each saliency condition (maximal or minimal). Before the analysis we made sure that our data did not violate the assumption of homoscedasticity. In line with our predictions, we expected an effect only for the regression model related to the maximal-saliency condition, indicating that a high-level of bottom-up sensory salience predicts the overall amount of successfully recollected information. More specifically, the first regression analysis assessed whether the probability of having or not having recollected the maximal-saliency object (recollection probability index) and the access at retrieval to the stored representation (recollection position index) predicted the contents of WM, i.e., the scaled amount of recollected objects for that given scene. In this regression model we used the recollection probability and the recollection position as predictors, and the scaled amount of recollected objects as dependent variable. Importantly, this approach (i.e., using two predictors instead of carrying out separate regression models) has the advantage of estimating the particular influence of each predictor while controlling for the influence of the other predictors at the same time. The second regression model was analogous to the first model, but now including the indexes related to minimal-saliency objects (again, the recollection probability and position as predictors, and the scaled amount of recollected objects as dependent variable). The data were analyzed with SPSS 13.0 (Statistical Package for Social Science, SPSS Inc.).

## Results

Overall, participants reported a mean of 5.0 objects across the scenes, with marked differences related to the recollection probability of maximal- and minimal-saliency objects, as highlighted in Figure 2A. A two-tailed paired-samples t-test revealed a significant difference for our participants in the probability of recollecting objects according to their saliency level (*t*_(19)_ = 15.4; *p* < 0.001), with maximal-saliency objects (0.66 ± 0.02) reported far more frequently than minimal-saliency objects (0.43 ± 0.02). Next, we analyzed whether perceptual saliency affected the position in which maximal- vs. minimal-saliency objects were recollected. As highlighted in Figure 2B, maximal-saliency objects (0.55 ± 0.01) were recollected earlier than minimal-saliency objects (0.65 ± 0.01; *t*_(19)_ = −6.8; *p* < 0.001), indicating that maximal-salient objects are prioritized during the recollection phase.

**Figure 3:**
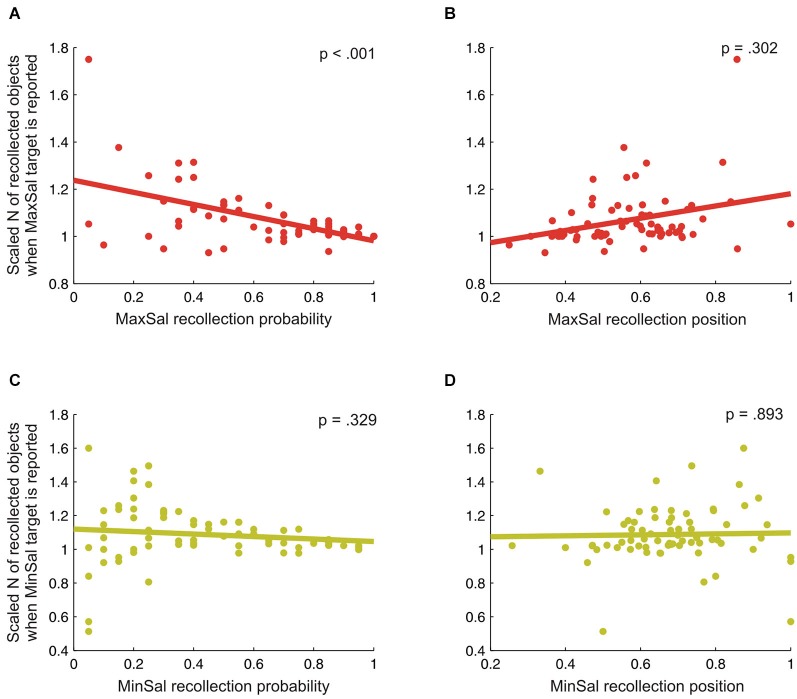
Scaled amount (N) of recollected objects when the maximal **(panels A and B)** or the minimal-saliency target **(panels C and D)** was successfully reported as a function of either the recollection probability **(panels A and C)** or the recollection position **(B and D)** of the target. Overall, these graphs indicate that the probability to have successfully recollected the maximal-saliency (MaxSal) object reduced the overall amount of recollected objects (cf. **panel A**).

The impact of perceptual saliency on free recollection of objects from natural scenes was further investigated by two regression analyses. These were used to establish whether increasing bottom-up sensory saliency at encoding (indexed by the probability to have successfully recollected maximal-saliency objects) and the specific access at retrieval to the stored representation of the scene (indexed by the recollection position) predicted the overall amount of successfully recollected objects (i.e., WM contents; see Melcher and Piazza, 2011). We found that, the current amount of successfully recollected information was modulated by perceptual saliency. The first regression model was significant (*F*_(2,70)_ = 15.9, *p* < 0.001, *R*^2^ = 0.319), and revealed a significant effect of the recollection probability index (*β* = −0.512, *t* = −4.7, *p* < 0.001; see Figure 3A) on the amount of recollected objects, but no effect of the recollection position index (*β* = 0.113, *t* = 1.0, *p* = 0.302; see Figure 3B).^2^ Then, the higher the probability of recollecting the maximal-salient object, the more the decrease in the amount of successfully recollected information. By contrast, the recollection position of the maximal-salience objects did not significantly predict the amount of successfully recollected information, indicating that the specific access to scene representation at retrieval did not affect the current contents of WM. The second regression model was instead not significant (*F*_(2,70)_ < 1, n.s., *R*^2^ = 0.015), indicating that neither the recollection probability (*β* = −0.126, *t* = −1.0, *p* = 0.329; see Figure 3C) nor the recollection position (*β* = −0.017, *t* = −0.1, *p* = 0.893; see Figure 3D) of minimal-saliency objects significantly predicted the amount of successfully recollected information.

## Discussion

The current study aimed to investigate whether low-level sensory features (i.e., bottom-up saliency) affected the probability of objects to be recollected from natural scenes, and, if so, whether the probability of recollecting maximal-saliency objects modulated the overall contents of WM. We presented pictures of natural scenes involving high-levels of competition among to-be-remembered objects. After an encoding phase of 4 s, and a retention phase of 8 s, we asked participants to verbally report as many objects as they could remember of the previous scene (i.e., a free-recollection task). It is worth noting here that free-recollection may suffer from potential limitations due to the involvement of other high-level cognitive functions, such as language (i.e., the requirement to “verbally” report the remembered objects). For instance, during the recollection phase a participant might fail to recall the “verbal label” (i.e., the name) corresponding to a specific object, with a consequent failure in verbally reporting that object, even though this was successfully stored into the internal memory representation. Crucially, however, here we did not make any specific assumption related to objects that were not recollected; by contrast, our analyses involved only those objects that were successfully recollected. Although we cannot assess whether objects that are not recollected are stored in memory or not, we must assume that a memory representation of the recollected objects exists. We therefore believe that the information related to successfully recollected objects in the current paradigm is reliable and can inform us about internal memory representation (or WM content) of those objects in the scene.

The current findings revealed that the probability of recollecting objects varied dramatically according to their saliency level at encoding (i.e., during scene viewing), with maximal-saliency objects reported far more often than minimal-saliency objects. We also found that the access to scene representation was facilitated for maximal-saliency objects, which were reported earlier in the stream of recollected items compared to minimal-saliency objects. Finally, we found that the probability to recollect maximal- (but not minimal-) saliency objects significantly predicted the overall amount of successfully recollected objects: the higher the probability of recollecting the maximal-saliency object, the lower the amount of recollected objects. Importantly, this effect was not significantly modulated by the current position of the target-object in the stream of reported items, indicating that this effect did not arise during access to the stored representation of the scene.

The increased memory performance (i.e., the recollection probability index) for maximal- vs. minimal-saliency objects is in line with the previous literature (Fine and Minnery, 2009; Santangelo and Macaluso, 2013). This effect is in agreement with the notion that bottom-up attention can modulate short-term memory, by increasing the likelihood of attentional “grabbing” items to be remembered later on (see, e.g., Schmidt et al., 2002; Botta et al., 2010). However, it is worth noting that here we use a more demanding WM task as compared to the previous literature (i.e., a free recollection task). Fine and Minnery (2009) used a task requiring a low-level of competition among the possible objects/targets, consisting on the encoding of only 3–5 items in each trial (i.e., not overloading WM capacity; Luck and Vogel, 2013). Santangelo and Macaluso (2013) used instead a task requiring a higher-lever of competition at encoding, presenting pictures of natural scenes (actually, the same as those used here), including a number of possible memory targets in each scene (i.e., a supra-span condition). However, Santangelo and Macaluso used at retrieval a visuo-spatial recognition test, presenting as memory target an object cut-out from the previous scene in the same or in a different position. This may have elicited responses simply based on a sense of “familiarity” with the scene. Here we use a more demanding WM task compared to this previous literature, that is a free recollection task (Craik and McDowd, 1987). As in all free recollection tasks (e.g., Lieberman and Culpepper, 1965), participants had no hints about the original scene (or—more generally—about the studied material), and they can only report what they had successfully encoded during scene viewing. The current finding therefore highlights that the saliency effect on memory performance is robust, revealing a prioritization on internal memory representation of maximal-saliency objects, over and above any sense of familiarity with the scene.

Bottom-up saliency not only increases the probability for an object to be recollected, but also speeds-up the access to the stored (memory) representation during the recollection phase. In fact, we found that maximal-saliency objects were recollected earlier than minimal-saliency objects in the stream of reported items. This prioritization effect at the retrieval phase is in line with recent findings reported by Pooresmaeili et al. (2014): they used a different paradigm wherein the salient/non-salient comparison was made at memory retrieval. Here we did not emphasize in any way one object above the others at encoding or retrieval (i.e., we used a “free” recollection task). Notwithstanding that, participants recollected the maximal-saliency object earlier than the minimal-saliency object. This might be interpreted in terms of a facilitated access (or a “prior entry”; see Spence and Parise, 2010) in the stored representation of the scene for objects located—during the encoding phase—at peaks of maximal-saliency.

Finally, the current findings highlight the role of perceptual saliency in affecting the overall number of objects successfully recollected from natural scenes. This finding might be interpreted in the light of the previous literature (Melcher and Piazza, 2011; see also Pooresmaeili et al., 2014), showing that bottom-up saliency affects the availability of WM resources, thus influencing its capacity. Accordingly, here we found that the higher the probability of reporting the most-salient object in the scene, the lower the overall amount of information successfully recollected in that scene. Although maximal-saliency objects are recollected on average earlier than minimal-saliency objects (see Discussion above and Figure 2B), the specific position of the maximal-saliency object in the stream of reported items did not affect the overall amount of recollected information (cf. the first regression model; see also red line in Figure 3B). In other words, the reduction in WM capacity was not modulated by the position of the target-object in the stream of recollected objects at retrieval. Although the interpretation of null effects has to be always very cautious, this finding (deserving further assessment in future research) seems to indicate that the decrease in the overall amount of successfully reported information did not arise during the attempt to access the information related to the target-object in the internal representation of the scene (see Pooresmaeili et al., 2014). By contrast, we suggest that during scene viewing (i.e., the encoding phase) the more an object is efficient to grab participants’ attention resources (according to its saliency level; see, e.g., Nardo et al., 2011, 2014), the less spared resources would be available to process other, lower-saliency, objects in the scene.

We acknowledge that the current task was not specifically designed to address the issue of whether the impact of perceptual saliency on WM contents arise at encoding or retrieval. In fact, we only collected WM performance at retrieval, without measuring any behavioral and/or physiological parameter during the encoding phase. Notwithstanding that, we note that our interpretation might be in good agreement with several models postulating an assignment of “attentional priorities” under conditions of high-levels of conflict/competitions among the stimuli (see, e.g., Desimone and Duncan, 1995; Itti and Koch, 2001; Pessoa, 2009). The assignment of attentional priorities might directly affect short-term memory representation (e.g., Bundesen, 1990). According to Bundesen et al. (2005, 2011), attention selection mechanisms would directly change the number of cortical neurons used to represent a given object, with the number of neurons increasing as a function of the task-relevance of the object itself. As a consequence, behaviorally important objects would have a high probability of winning the competition to be encoded and thus accessing an internal representation through the short-term memory system. The latter is conceived as a feedback mechanism that sustains activity in the neurons that have won the attentional selection/competition (see also Cowan, 1995, 2011, for a similar notion). Here we used a task in which all objects in the scene were equally task-relevant. In fact, we asked participants to freely report all objects they could remember. Notwithstanding that, we showed that objects corresponding to the point of maximal (vs. minimal) saliency in the scene were recollected with higher probability (reducing at the same time the overall amount of information successfully reported). This is consistent with the notion that visual saliency plays a key role in assigning attentional priorities (see, for reviews, Thompson and Bichot, 2005; Gottlieb, 2007). Speculatively, we interpret our findings within the framework of Bundesen et al.’s theory: the most-salient object would receive attentional resources and then encoding priority; this would lead to a higher recollection probability for the most-salient objects, but also to less attention resources for the other (lower saliency) objects in the scene, consistent with the reduction in the overall amount of information successfully recollected (cf. Figure 3A).

To conclude, the current study provided initial evidence that the processing of a maximal-saliency object in a natural scene is prioritized during formation of objects/scene memory traces and during later access to this stored representation. We found a reduction of the overall amount of successfully recollected information when maximal-salience objects entered the internal memory representation, thus having a higher chance to be recollected later on. We interpreted such a reduction as evidence that high-level perceptual saliency tends to exhaust attentional resources during the exploration of a natural and complex scene.

## Conflict of interest statement

The authors declare that the research was conducted in the absence of any commercial or financial relationships that could be construed as a potential conflict of interest.

## Footnotes

^1^http://www.saliencytoolbox.net/

^2^Following the inspection of Figure 3A, one might argue that this regression analysis could be affected by an outlier data point (cf. the red dot in the upper-left corner). However, the regression model was still significant after removing this data point (*F*_(2,69)_ = 12.4, *p* < 0.001, *R*^2^ = 0.269), showing again a significant effect of the recollection probability index (*β* = −0.506, *t* = −4.5, *p* < 0.001) on the amount of recollected objects, but no effect of the recollection position index (*β* = 0.036, *t* = 0.3, *p* = 0.748).
